# Comparison of the Antioxidant Properties of Green Macroalgae from Diverse European Water Habitats by Use of Several Semi-Quantitative Assays

**DOI:** 10.3390/molecules27123812

**Published:** 2022-06-14

**Authors:** Zuzanna Piotrowicz, Łukasz Tabisz, Bogusława Łęska, Beata Messyasz, Radosław Pankiewicz

**Affiliations:** 1 Faculty of Chemistry, Adam Mickiewicz University, Uniwersytetu Poznańskiego 8, 61-614 Poznań, Poland; zuzpio1@amu.edu.pl (Z.P.); lt35808@amu.edu.pl (Ł.T.); bogunial@amu.edu.pl (B.Ł.); 2 Department of Hydrobiology, Faculty of Biology, Adam Mickiewicz University, Uniwersytetu Poznańskiego 6, 61-614 Poznań, Poland; messyasz@amu.edu.pl

**Keywords:** polyphenols, chlorophyll, *Ulva*, *Cladophora*, macroalgae, Chlorophyta, colorimetric assays, ABTS, FRAP, Folin–Ciocalteu, complexometric with Al^3+^ ions

## Abstract

Nowadays, algae are becoming more and more popular as a food group rich in nutrients, cosmetic raw materials full of antioxidants or valuable dietary supplements. They are of interest for the industry because they are found almost all over the world, in all climatic zones, both in fresh and salt waters. The aim of this study was to take a broad look at green algae (Chlorophyta) and to show how large the variability of the content of active compounds may depend on the species and the place and time of sample collection. Particular attention was paid to compounds with antioxidant activity, whose simplified profiles were created on the basis of complementary, semi-quantitative methods. Additionally, time-yield extraction optimizations were performed. Three different specimens of *Ulva lactuca* were compared: from the coastal zone of the Baltic Sea, from the open Baltic Sea area around Bornholm and *Ulva spiralis* (*Ulva lactuca* polymorph) from the Atlantic Ocean. The studied algae of the *Cladophora* genera were three different species of freshwater algae from various habitats: a lake (*Cladophora glomerata*), a river (*Cladophora rivularis*) and aquarium farming (*Cladophora aegagropila,* syn. *Aegagropila linnaei*). The content of antioxidants and the extraction efficiency varied significantly depending on the species.

## 1. Introduction

Growing human population means that we will, sooner rather than later, have to face the problem of finding new sources of nutrients. Moreover, in developed countries, much attention is paid to a healthy lifestyle, which includes a balanced diet, prevention of civilization diseases and premature aging. In response to the above, food and cosmetic industries have shown increasing interest in raw products, that are rich in antioxidants and that would be able to meet the increasing expectations of consumers. One such raw product seems to be algae, which are enjoying increasing popularity as a food group rich in valuable nutrients, cosmetic raw products rich in antioxidants or valuable diet supplements [1,2].

A definite advantage of algae is that they occur all over the world, in all climate zones, and in fresh as well as salty waters. They are autotrophic organisms of low living requirements, occurring in a diversity of forms: from unicellular microalgae as freely flowing phytoplankton, to macroalgae forming thalli of various sizes, some of them resembling vascular plants. Both micro- and macroalgae may be collected from their natural habitats or grown in coastal farms (macroalgae) or in special containers (microalgae) [3,4]. Low living requirements and fast biomass growth make algae a valuable raw material for industry (biofuels, animal feed, dietary supplements, etc.) [5,6]. They contain relatively large amounts of iodine; vitamins A, C, B3 and B9; polysaccharides; simple sugars; dyes; proteins and amino acids, and some species are even rich in fatty acids [7]. From the point of view of our studies, they are a source of polyphenols showing in vitro antioxidant effect [8]. Antioxidants are important components of a balanced diet and essential agents active against civilization diseases, including diabetes, cardiovascular diseases or neurodegenerative diseases [8,9,10,11,12]. Antioxidant compounds present in macroalgae, such as phenols, flavonoids, alkaloids, tannins, pigments and others, play an important role in the human organism, supporting its defense reactions against free radicals (ROS) and the effects of their destructive action (such as DNA damage). Thanks to this, algae extracts can be used in the prevention of civilization diseases, such as various types of cancer [2].

The main objective of this study was to compare the two genera of multicellular macroalgae from the division of green algae (*Ulva* and *Cladophora*), originating from different habitats, as to the content of phenolic compounds and chlorophyll, which, as previously reported [13], also acts as free radical scavenger. By studying extracts of six green macroalgae (living either in freshwater or marine water, representing species from different habitats) using a few mutually complementary methods for determination of antioxidant activity, it was possible to create a simplified profile of the antioxidant types in each of the tested species. Moreover, the process of extraction was optimized, taking into account the extraction yield and time, while testing three variants of continuous extraction: in a Soxhlet apparatus, in a Knöfler-Böhm apparatus and Soxhlet extraction preceded by preliminary ultrasound-assisted extraction.

The samples of algae selected for the study are characterized in literature as the most abundant and ubiquitous in water ecosystems around the world [14,15]. The first marine species, *Ulva lactuca* L. (UL), belongs to Chlorophyta (green algae) identified in the Baltic Sea and was described by Linnaeus in the 17th century [16]. It is a polymorph and may assume different forms depending on the degree of water salinity [17] or symbiosis with bacteria [16]. It may freely flow in seawater or grow anchored to the sea bottom, stones or pier logs; usually it looks like a lettuce. According to genetic analyses, other green algae of tubular phenotypes are the clads of *U. lactuca*, although they have been earlier described as different species or even genera, like *Ulva lactuca* var. *spiralis* (Hornemann) Lange (UI), used in this study [18].

*Ulva lactuca* is characterized by a high content of soluble and insoluble fiber (jointly they make 54% of dry mass); minerals (calcium, potassium, sodium and phosphorus making 9.6%); proteins (8.5%) including aspartic acid, glutamic acid, alanine, valine and leucine; fats (7.9%) including mainly palmitic acid (60%) and oleic acid (16%) [19]; polysaccharides, with mainly sulfated polysaccharides (12.80—30% of all polysaccharides); and rhamnose, xylose and uronic acid [20]. *Ulva* also has a high content of tocopherols [21], which show antioxidating properties, similar to phenols. No literature data have been found detailing the content of polyphenols in this species of algae. Literature is lacking when it comes to the precise phenolic composition of *Ulva lactuca* species, but some authors mention that it contains, among others, such phenols as caffeic acid, chlorogenic acid, ferulic acid, *p*-coumaric acid, salicylic acid, protocatechuic acid, pyrogallic acid, resorcinol, vanillin [22], L-tyrosine and L-phenylalanine [23].

*Cladophora* has reticulated filamentous branched thallus, often resembling a bush. *Cladophora* represents a cosmopolitic genus, found in freshwater and marine water, and it may show great variation in appearances and chemical compositions, depending on habitat and environmental conditions. Because of the high content of proteins, fiber and vitamins, *Cladophora* has been used as food since antiquity [24]. *Cladophora glomerata* (L.) Kützing (CG) is a widespread filamentous algae, occurring in a broad range of water ecosystems (habitats), including lakes, rivers, streams and tidal zones [25]. It is one of the most common species in the Baltic Sea [26]; its characteristic features are rapid growth of biomass and formation of large surface clusters. It is also a rich source of biologically active compounds, including saturated and unsaturated fatty acids, sterols, terpenoids, phenolic compounds [27] and proteins (about 14% of dry mass) [28]. Another algae studied, which represents the family *Cladophoraceae,* is *Cladophora rivularis* (L.) Kuntze Hoek (CR). It also is a cosmopolitic filamentous green alga of rich chemical composition, including hydrocarbons, phenols, aldehydes, terpenoids and sterols, such as cholesterol, sitosterol, 24-methylcholesta-5,24(28)-dien-3B-ol and fatty acids, mainly palmitoleic acid, palmitic acid, oleic acid and myristic acid [29]. The last macroalgae species selected for testing in this paper is *Cladophora aegagropila* (L.) Trevison (syn. *Aegagropila linnaei* Kützing) (CA) also known as spherical seaweed or marine moss, because of its characteristic shape resembling a green ball made of filamentous thallus due to the water waves effect. It is found in shallow lakes, mainly in Eurasia, but also in a part of Japan where it is under strict protection. The most impressive specimens may be found in Lake Baykal in Russia [30]. Because of its attractive look, this algae species is often used as an ornamental plant in aquariums. There are no literature data on its chemical composition, it is only known to contain significant amount of carotenoids [31,32,33]. Moreover, only a single study has been undertaken to determine the impact of four organic compounds: ascorbic acid, biotin, glucose and sucrose, on ash, protein, fiber, fat and amino acid content in the freshwater *Aegagropila linnaei* biomass [34].

In this paper, we present data on antioxidant properties of extracts obtained from green macroalgae (*Cladophora*, *Ulva*), collected from diverse European water habitats, with the intent of describing and interpreting the range of variation of biologically active compounds. A simplified antioxidant profile was elucidated on the basis of complementary semiquantitative colorimetric methods. Moreover, the contents of chlorophyll in the thalli of all studied algae were determined.

## 2. Results and Discussion

### 2.1. Optimization of Extraction Process

**Figure 1:**
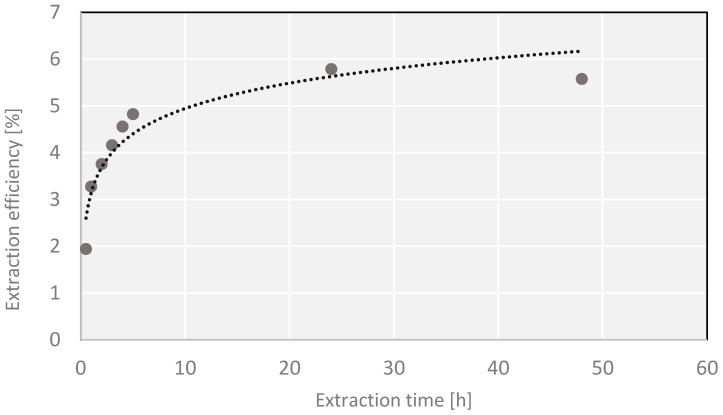
Dependence of the yield on the extraction time in a Soxhlet apparatus.

No significant differences in the extraction yield of the processes carried out by different methods were observed when the process was carried out for 24 h. Slightly higher yields favor the use of a simple Soxhlet apparatus (Figure 1).

**Figure 2:**
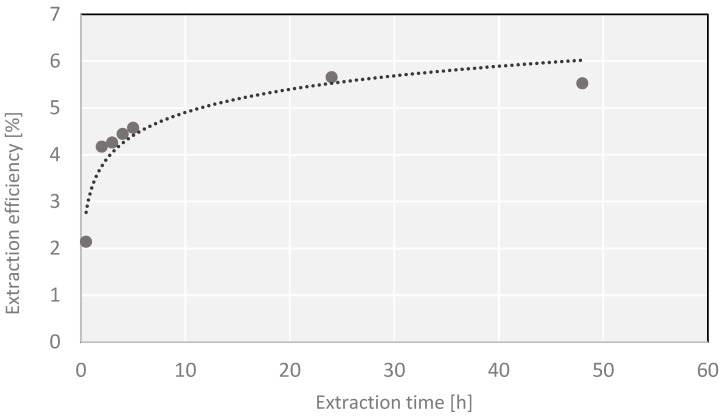
Dependence of the yield on the extraction time in a Knöfler-Böhm apparatus.

**Figure 3:**
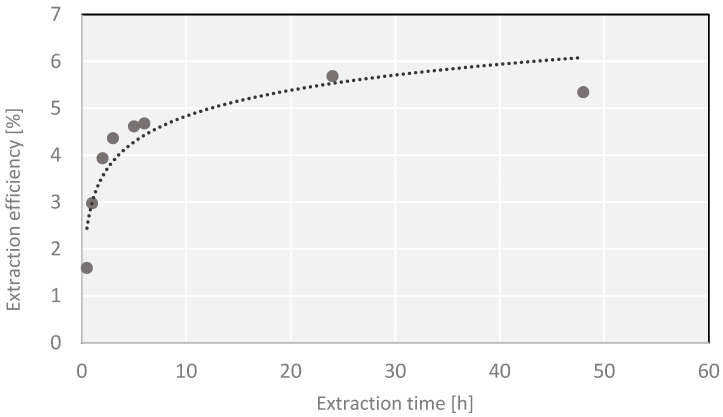
Dependence of the yield on the extraction time in an ultrasonic bath and Soxhlet apparatus.

The experiments also pinpointed the same time, 24 h, as the duration needed for isolation of all active substances that can be extracted with a medium-polarity solvent (ethanol). Longer time of extraction leads to decreasing extraction yields, probably owing to the decomposition of thermally labile chemical compounds or the loss of more volatile substances. When it is necessary to shorten the extraction time below 24 h, the results obtained by extraction in a Knöfler-Böhm apparatus seem attractive (Figure 2). For the extraction time of up to 3 h, the process in a Knöfler-Böhm apparatus is more efficient than in a Soxhlet one. It is probably a consequence of the difference in construction of the two extractors. In the Soxhlet apparatus, the process is carried out with a solvent whose temperature is much lower than its boiling point, while in the Knöfler-Böhm apparatus, hot vapor of the solvent heats the whole extraction vessel so the extraction is performed with a temperature very close to the solvent’s boiling point. As follows from our results, this small difference has a significant impact on the amount of the substance extracted in the initial phase of the process. The application of preliminary ultrasound-assisted process prior to the main one did not bring the expected effects (higher yields) (Figure 3) as suggested by some authors [35].

### 2.2. Yields of Extractions

**Figure 4:**
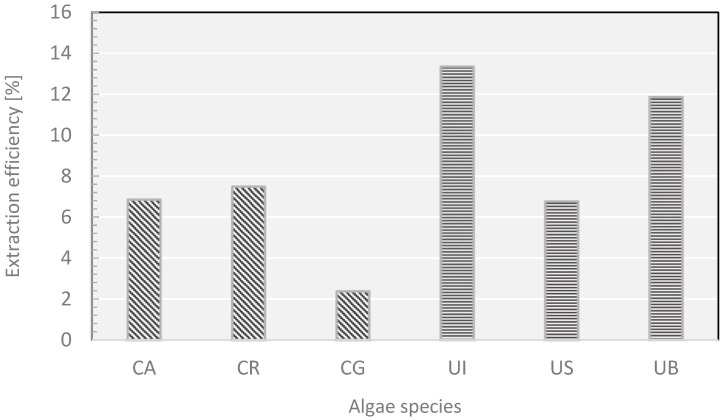
Percentage extraction efficiency of individual algae samples.

The active compounds extracted from algal thalli may depend on the type of algae material, site and time of collection (which determines growth parameters, such as temperature, sunlight exposure, water pH and salinity) [36]. Moreover, the yield of extraction (per dry mass) depends also on the presence of encrustations, mainly calcium carbonate [37], and epiphytes-other algae species, mainly microalgae [25,38]. Although a comparison of biomass growth of green algae from different water habitats was not the main aim of this study, it is worth noting how many different factors have a significant impact on the yield of extractions. The scale of the difference is best seen by comparing the extract yields (Figure 4) from *Cladophora glomerata* (CA)—used both for the optimization of the extraction process and as a source of extract for semi-quantitative determination of antioxidants—which was collected for those purposes at the same site, but at different times. Most probably the differences (5.79% vs. 2.38%) stem not only from the variations in biomass accumulation, but also from variations in the presence of incrusts and epiphytes.

The latter may vary not only within the vegetation season, but also from year to year, according to a mechanism that is not yet fully established. In order to explain the low yield of *Cladophora glomerata* (CA) extraction, a sample of this algae was subjected to de-incrustation. The weighted sample of the algae was carefully washed with a solution of acetic acid, dried and weighted again (the procedure is described in detail in [37]). After elimination of incrusts, composed mainly of calcium carbonate, the dry material lost nearly 45% of its weight. It is reasonable to suppose that their presence was responsible for the much lower yield of extraction of *Cladophora glomerata* (CA).

### 2.3. The Content of Chlorophyll

**Figure 5:**
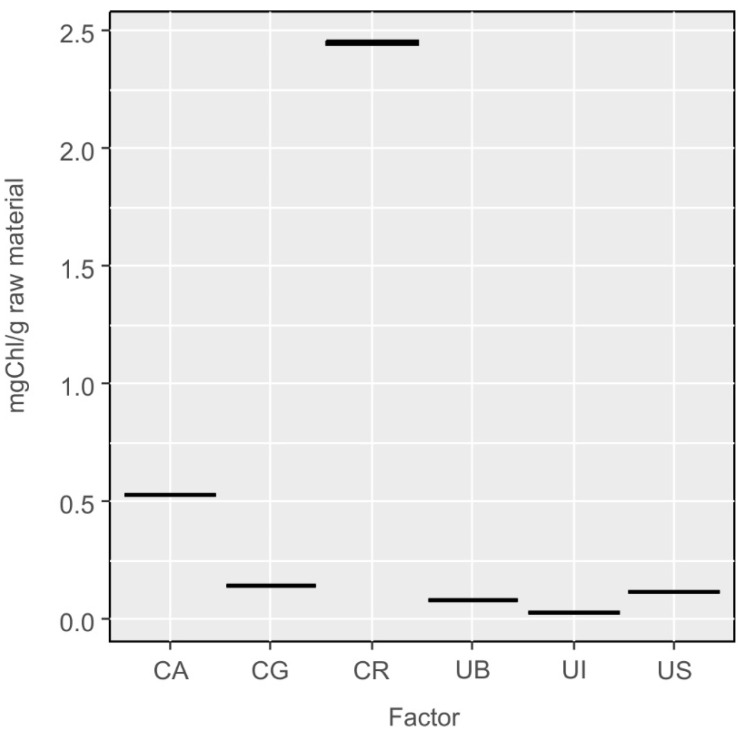
Results of determinations of the total content of chlorophyll in the extracts from *Ulva* and *Cladophora* species, converted into milligram of chlorophyll per gram of raw material.

The content of chlorophyll in algae directly depends on the thallus’s access to sunlight during the day; when the exposure is lower, the algae need to produce a greater amount of chlorophyll to ensure the supply of sufficient energy to be accumulated in the bonds of chemical compounds produced in the photosynthesis. This dependence is well seen for the samples of *Ulva*—the extracts from the samples collected near Sopot (US), where the water is turbid and of low transparency, contained larger amounts of chlorophyll, while the samples collected from the Atlantic Ocean (UI), where the water is clear, had a much lower content of chlorophyll (Figure 5).

For the freshwater algae, the highest content of chlorophyll was found in *Cladophora rivularis* (CR), which grows on the river bottom at sites often shadowed by coastal vegetation. The lowest content of chlorophyll was determined in the extracts of *Cladophora glomerata* (CA) collected from a lake where it floated freely under well-sunlit water surface (Figure 5). One-way ANOVA analyses of the differences in chlorophyll content between all species were statistically significant F_5,12_ = 415, *p* < 0.001. The statistical differences were related to all species from *Ulva* and *Cladophora* at the significance level *p* < 0.001 for the Tukey HSD test.

### 2.4. Antioxidative Potential

**Figure 6:**
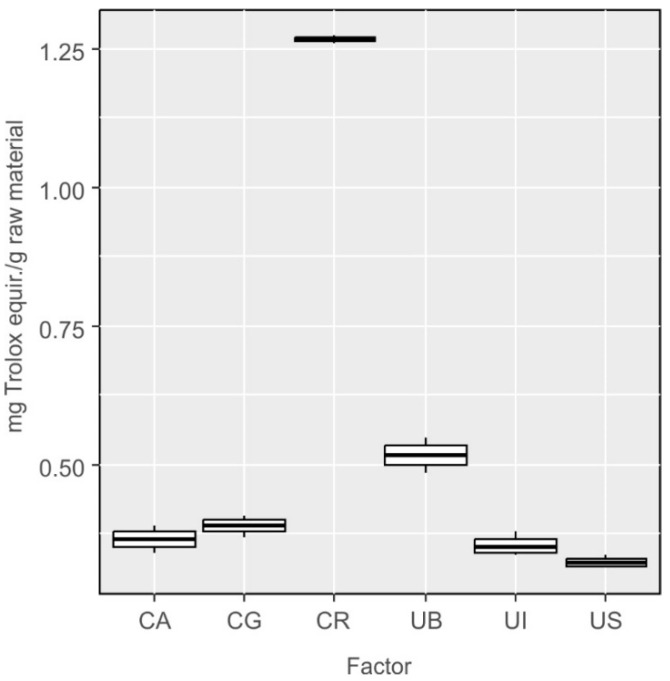
Results of determination of the content of compounds with antioxidant activity in extracts from *Cladophora* and *Ulva* species using the ABTS method, converted into milligram equivalents of trolox per gram of raw material.

By using ABTS assay, it is possible to semi-quantitatively determine the total content of antioxidants in a given sample (extract) (Figure 6). Statistical analyses confirmed that the differences in the total content of antioxidants in some species were statistically significant (One-way ANOVA: F_5,12_ = 881.7, *p* < 0.001). The differences were not statistically significant between the following species: *Cladophora glomerata* and *Cladophora aegagropila* (CG-CA, *p* = 0.70), *Ulva lactuca* var. *spiralis* and *Cladophora aegagropila* (UI-CA, *p* = 0.96), *Ulva lactuca* from Sopot and *Cladophora aegagropila* (US-CA, *p* = 0.19), *Ulva lactuca* var. *spiralis* and *Cladophora glomerata* (UI-CG, *p* = 0.299) and *Ulva lactuca* from Sopot and *Ulva lactuca* var. *spiralis* (US-UI, *p* = 0.529) (Figure 6).

**Figure 7:**
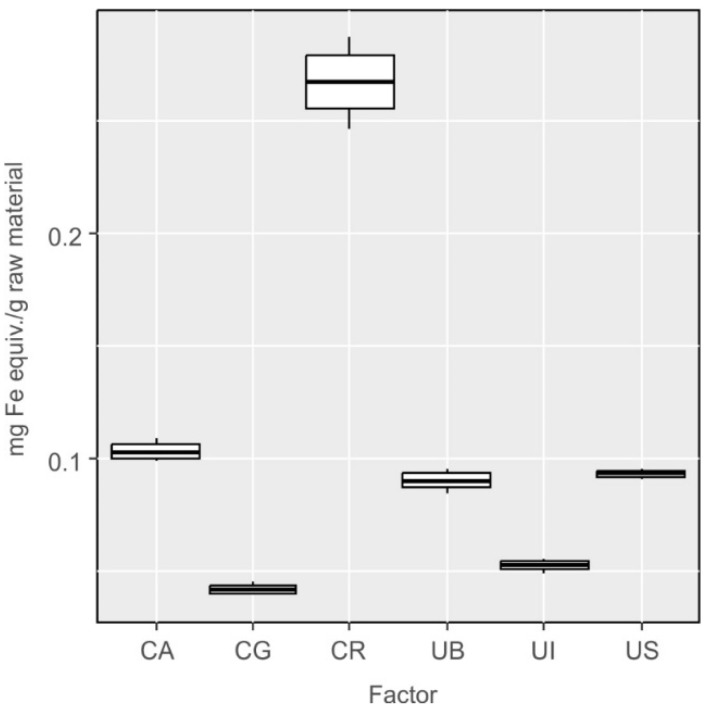
Results of determination of the content of compounds with antioxidant activity in extracts from *Cladophora* and *Ulva* species using the FRAP method, converted into milligram equivalents of iron per gram of raw material.

Unfortunately, the method is not highly specific for phenolics, and the results may be falsified when measurements are performed in the presence of carotenoids, as well as vitamin E and C. For this reason, verifying measurements are recommended by the FRAP method, which is insensitive to the presence of the latter compounds (Figure 7). Again, it was found that the differences in the content of these components between investigated species were statistically significant (F_5,12_ = 235.7, *p* < 0.001). The differences were not statistically significant between the following species: *Ulva lactuca* from Bornholm and *Cladophora aegagropila* (UB-CA, *p* = 0.55), *Ulva lactuca* var. *spiralis* and *Cladophora aegagropila* (UI-CA, *p* = 0.96), *Ulva lactuca* from Sopot and *Cladophora aegagropila* (US-CA *p* = 0.78), *Ulva lactuca* var. *spiralis* and *Cladophora glomerata* (UI-CG, *p* = 0.72) and *Ulva lactuca* from Sopot and Bornholm (US-UB *p* = 0.99) (Figure 7).

Comparative analysis of the results obtained by these two methods provides the information on the approximate content of carotenoids in the extracts studied. The importance of making measurements by these two methods is particularly pronounced for the samples of *Ulva lactuca* var. *spiralis* (UI) and *Ulva lactuca* (US) from Sopot and *Cladophora glomerata* (CA); their ABTS results indicate high content of antioxidants, while their FRAP data point to much lower content of antioxidants. This observation leads to a conclusion that these samples contain significant amounts of non-phenolic chemical compounds that show antioxidant activity. By far the richest in antioxidants was *Cladophora rivularis* (CR), which was indicated by the results obtained by both methods. It should be mentioned that the total yield of extracted compounds does not necessarily imply the highest content of antioxidants. For the samples of *Ulva lactuca* var. *spiralis* (UI) and *Ulva lactuca* (UB) from Bornholm the highest extraction efficiency was obtained, but their results of ABTS and FRAP assays show that they in fact have low antioxidant content. This result suggests that these samples have to be rich in other chemical compounds not detectable by these two methods.

**Figure 8:**
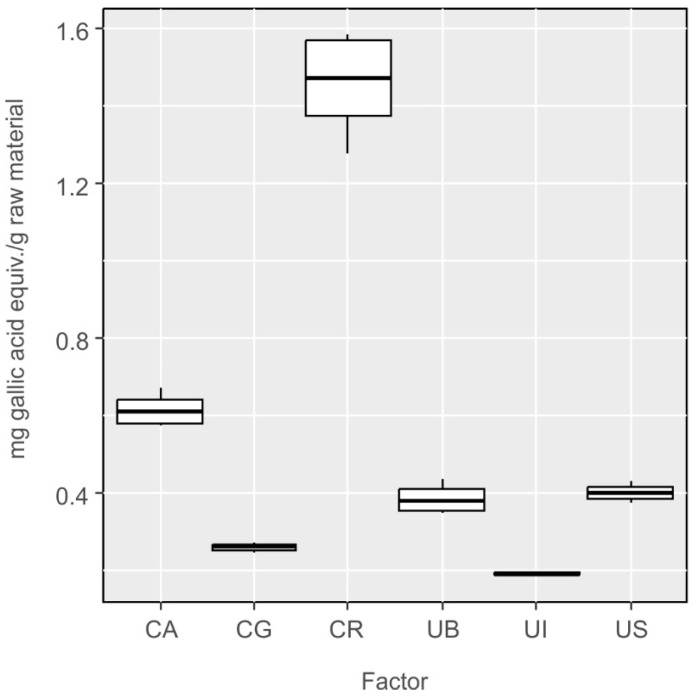
Results of determination of the content of polyphenols in extracts from *Cladophora* and *Ulva* species using the Folin–Ciocalteu method, converted into milligram equivalents of gallic acid per gram of raw material.

From among the samples of algae representing the *Ulva* species, the greatest content of phenolic compounds was found by the Folin–Ciocalteu method in the samples collected near Sopot, while the lowest was found in the algae collected near Bornholm (Figure 8). One-way ANOVA of the differences between investigated species was statistically significant with F_5,12_ = 235.7, *p* < 0.001. The differences were not statistically significant only in four cases: *Ulva lactuca* from Bornholm and *Cladophora glomerata* (UB-CG, *p* = 0.40), *Ulva lactuca* var. *spiralis* and *Cladophora glomerata* (UI-CG, *p* = 0.87), (US-CG, *p* = 0.26) and *Ulva lactuca* from Sopot and Bornholm (US-UB, *p* = 0.99) (Figure 8).

Sopot lies at the Gdansk Bay whose waters are turbid, strongly polluted and relatively shallow. The state of the water in the bay may stimulate the algae to produce greater amounts of phenolic compounds in response to the environmental stress. The water of the Atlantic Ocean, from where *Ulva lactuca* var. *spiralis* (UI) was collected, is clear and transparent, providing the optimum conditions for algae growth and biomass accumulation, so it is understandable that under no environmental stress, less phenolic compounds are produced. In the samples of *Cladophora*, the lowest content of phenolic compounds was determined in *Cladophora glomerata* (CG) collected from the lake, where the algae thalli float directly under the surface and have unlimited access to oxygen and sunlight. These conditions and low environmental stress are responsible for a low content of polyphenols. In contrast, *Cladophora rivularis* (CR) growing in the river showed higher content of polyphenols probably in response to high anthropopressure.

**Figure 9:**
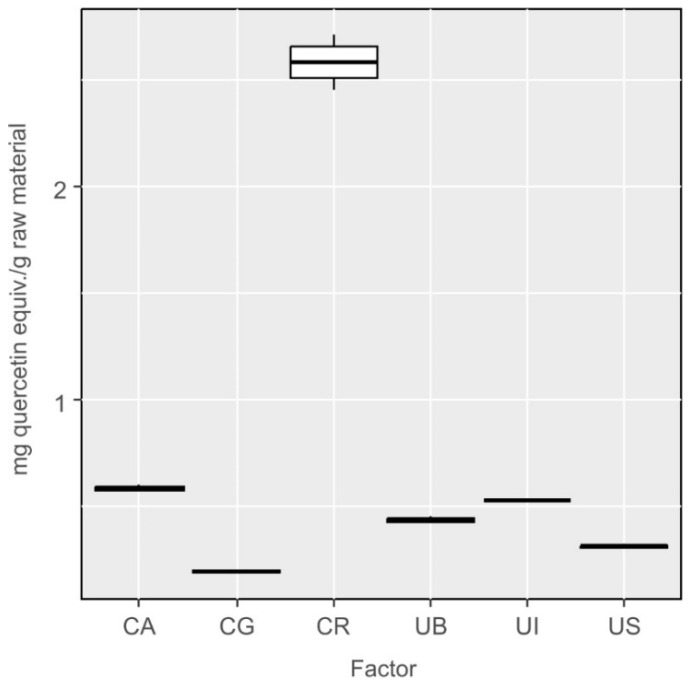
Results of determination of the content of flavonoids in extracts from *Cladophora* and *Ulva* species using the complexometric with Al^3+^ ions method, converted into milligram equivalents of quercetin per gram of raw material.

Results of complexometric method with Al^3+^ ions permitted estimation of the fraction of flavonoids, a subdivision of phenolics. According to the results obtained for *Ulva* species (Figure 9), it may be concluded that the specimens collected near Sopot show relatively low content of flavonoids, whereas flavonoids make a substantial fraction of polyphenolic compounds in the specimens of *Ulva lactuca* var. *spiralis* (UI), collected near Ireland. Statistical analyses confirmed that the differences in the content of flavonoids between species were statistically significant (One-way ANOVA: F_5,12_ = 863.1, *p* < 0.001). The differences were not statistically significant between the following species: *Ulva lactuca* var. *spiralis* and *Cladophora glomerata* (UI-CA, *p* = 0.79), *Ulva lactuca* from Sopot and *Cladophora glomerata* (US-CG, *p* = 0.12), *Ulva lactuca* var. *spiralis* and *Ulva lactuca* from Bornholm (UI-UB, *p* = 0.30) and *Ulva lactuca* from Sopot and Bornholm (US-UB, *p* = 0.12) (Figure 9). In the samples of *Cladophora* algae, the contents of flavonoids and the other polyphenols were comparable.

An attempt was made to correlate the results obtained with each of the methods, but unfortunately, due to large differences in standards, it was not possible to find a mathematical formula that would allow for appropriate conversions.

### 2.5. Impact of Environment

The sample of *Cladophora aegagropila* (*syn. Aegagropila linnaei*) was collected from aquarium farming, with a constant and controlled living conditions, so the results obtained for this sample were treated as a reference. The studied sample of *Cladophora glomerata* was collected from the lake where this species had good living conditions, so the sample contained low amounts of chlorophyll and phenolic compounds. The studied sample of *Cladophora rivularis* was collected from the river Nielba, offering quite different living conditions; the algae grew in the water flowing in a narrow and shaded channel, so the development of their thalli was delayed. The extract from this sample contained high amounts of chlorophyll and phenolic compounds, which are related to allelopathy in the conditions of growing competition. The extracts from the samples of Ulva collected near Bornholm and Ireland were very similar and probably contained large amounts of lipids and a relatively low content of chlorophyll. The algae at these two sites developed in the conditions of low competition, in cool, open water. The extract from the algae collected near Sopot showed large content of chlorophyll and phenolic compounds, the latter of which may indicate high competition. The algae grew in shallow water of high turbidity.

## 3. Materials and Methods

### 3.1. Algae Studied

Six samples of green algae collected from different habitats were studied. *Cladophora glomerata* (L.) Kützing was collected manually from Lake Oporzyn in Wagrowiec, near Poznan, Poland (52′55″ N, 17′9″ E). *Cladophora rivularis* (L.) Kuntze was collected from the river Nielba, also in Wagrowiec (52′48″ N, 17′12″ E). *Cladophora aegagropila* (L.) Trevisan (syn. *Aegagropila linnaei* Kützing) came from aquarium farming. Two samples of *Ulva lactuca* were studied, one collected from the Baltic Sea coastal zone, protected from sea currents (Gdansk Bay), near strongly urbanized area of the city of Sopot, Poland (54′45″ N, 18′57” E), and the other coming from the shores of Bornholm Island, in the southwestern part of the Baltic Sea (55′08” N, 14′69” E). The sample of *Ulva lactuca* var. *spiralis* (Hornemann) Lange (Irish spirulina) originated from a commercial source (AlgAran Teoranta; Kilcar, Co. Donegal, Ireland; 54′54” N, 8′37” W).

### 3.2. Reagents

Ethanol was used as the extraction solvent (96%, special purity, Avantor, Gliwice, Poland). Other solvents were of analytical or higher grade (Merck, Darmstadt, Germany). In addition, the following reagents were used in colorimetric assays: Folin–Ciocalteu’s reagent (analytical grade; Merck), sodium carbonate (>99%; Avantor), potassium persulfate (>99%; Merck), 2,2′-azino-bis(3-ethylbenzothiazoline-6-sulfonic acid) diammonium salt (ABTS;  >98%; Merck), 2,4,6-tris(2-pyridyl)-s-triazine (>98%; Merck) and Al(NO_3_)_3_·9H_2_O (>99.9%; Merck); the following were used as reference standards: gallic acid (>99%; Merck), quercetin (≥95% by HPLC; Merck) and 6-hydroxy-2,5,7,8-tetramethylchroman-2-carboxylic acid (“Trolox”; >97%; Merck). All reagents were purchased from Merck.

### 3.3. Methodology

#### 3.3.1. Optimization of Extraction Process

A series of extractions of *Cladophora glomerata* material were performed, lasting for 0.5, 1, 2, 3, 4, 5, 6, 24 and 48 h. Samples of ~15 g of dry mass were ground, placed in a cellulose thimble and subjected to continuous extraction with 300 mL of ethanol. The extraction was carried out in a 250 mL Soxhlet apparatus. The warm extracts were filtered and evaporated under reduced pressure to a constant mass. The yields of the processes for different times of extraction were compared. Similarly, the process of extraction was also performed in a 250 mL Knöfler-Böhm apparatus, following the procedure analogous to that of the extraction in a Soxhlet apparatus. In order to check the effects of ultrasounds on the efficiency of extraction, a series of ultrasound-assisted extractions were applied as preliminary treatment prior to a later, proper extraction in a Soxhlet apparatus. In order to perform ultrasound-assisted extraction, a portion of ~15 g of dry mass of *Cladophora glomerata* was weighted, ground, placed in a pure round-bottomed flask and flooded with 300 mL of ethanol. The extraction was carried out in an ultrasonic bath (Sonic 10, Polsonic, Warszawa, Poland) at 40–45 °C for 30 min. Then, the whole sample was moved to a cellulose filter thimble and the process was continued in a Soxhlet apparatus with the same solvent. The total extraction times were the same as in the first procedure. Further procedure steps were the same as in the process without ultrasound pretreatment.

#### 3.3.2. Extraction in a Soxhlet Apparatus

After optimization of the process, the extractions were performed in a Soxhlet apparatus, without pretreatment. The same procedure was followed, but due to limited sample sizes from the specimens of *Cladophora rivularis*, *Ulva lactuca* from Sopot and Bornholm and *Ulva lactuca* var. *spiralis*, and extracts were obtained from samples of 2.65 g, 0.62 g, 1.03 g and 10.54 g. The extractions were carried out for 24 h, then the samples were filtered at elevated temperature and evaporated under reduced pressure until a constant mass was reached. 

#### 3.3.3. Semi-Quantitative Colorimetric Assays

The contents of antioxidants in the extracts were determined by semi-quantitative colorimetric assays: ABTS, FRAP, Folin–Ciocalteu and complexometric method with Al^3+^ ions. Previously described methodologies were followed [39]. In some cases, further the dilutions of methanol extracts were necessary as specified in the table in Supplementary Materials. When applying the ABTS method and the complexometric method with Al^3+^ ions, mixing of the reagents with methanol solutions of algae extracts resulted in significant turbidity, so these methods had to be modified. One of possible reasons for the appearance of turbidity was a high content of lipophilic components. Elimination of water from the system and replacing it with methanol resolved the problem of turbidity. Additionally, to ensure high clarity of the solutions, the methanol solutions of algae extracts were centrifuged at 12,000 rpm for 5 min.

#### 3.3.4. Determination of the Content of Chlorophyll

The content of chlorophyll in the algae extracts was determined by the method described by Catarina Osório et al. [40]. Methanol solutions of dry algae extracts of a concentration of 1 mg/mL were made. The exception was the extract from *Ulva spiralis*, which was dissolved in methanol to the concentration of 10 mg/mL because of a low content of chlorophylls. In contrast, the extract of *Cladophora rivularis* had to be diluted to the concentration of 500 µg/mL. The absorbances of the solutions were measured at two wavelengths: 652 and 665 nm. The content of chlorophylls in the samples was calculated from the following formulas:

Chlorophyll a (μg/mL) = 16.72(A665) − 9.16(A652)

Chlorophyll b (μg/mL) = 34.09(A652) − 15.28(A665)

All determinations were performed on a Jenway 7415 spectrophotometer (Cole-Parmer, Saint Neots, UK).

### 3.4. Statistical Data Analysis

Statistical analyses were made with the R software. To test differences in chemical parameters between species we used one-way ANOVA. When the model was significant (*p* < 0.05), we used Tukey HSD test as post hoc.

## 4. Conclusions

The use of three mutually complementary semi-quantitative methods for determination of antioxidants allows for establishing of a simplified phytochemical profile of selected green algae. Two groups of algae were studied: the marine algae representing the species *Ulva* and the freshwater algae representing the species *Cladophora*. The samples of the algae were collected at different locations, and in order to check a possible correlation, the results of determinations of the active compounds in the thalli (in particular antioxidants) with the character of the habitats. The phytochemical profile was supplemented with determination of the chlorophyll content in algal extracts. The obtained results clearly show that the habitat and its physicochemical conditions have a significant impact on the content of polyphenols and photosynthetic pigments in the algae thalli. Thus, this method can be used to roughly assess the condition (high or low amount of oxidative stress) of the environment from which algae samples were collected.

The total yield extracted with a given solvent does not necessarily translate into a proportional content of antioxidants. Therefore, some extracts may be rich in other, non-phenolic compounds, and soluble in moderately polar solvents such as ethanol. Through optimization of extraction conditions, it was proved that all desired active compounds extractable with a given solvent were actually isolated. The classical extraction in a Soxhlet apparatus was found to be the most effective, and the optimum time of extraction was found to be 24 h (maximum yield without decomposition of thermally labile chemical compounds).

## Data Availability

Not applicable.

## Supplementary Materials

The following supporting information can be downloaded at: https://www.mdpi.com/article/10.3390/molecules27123812/s1, Figure S1: Results of determination of the content of compounds with antioxidant activity in extracts from algae using ABTS method, Figure S2: Results of determination of the content of compounds with antioxidant activity in extracts from algae using the FRAP method, Figure S3: Results of determination of the content of polyphenols in extracts from algae using the Folin-Ciocalteau method, Figure S4: Results of determination of the content of flavonoids in extracts from algae using the complexometric with Al^3+^ ions method.

## Sample Availability

Samples of the compounds are not available from the authors.
