# Supplementary material for: Comparison of the Antioxidant Properties of Green Macroalgae from Diverse European Water Habitats by Use of Several Semi-Quantitative Assays
Source: Molecules. 2022 Jun 14;27(12):3812. doi: 10.3390/molecules27123812 (PMC9227508; doi:10.3390/molecules27123812)
Supplement: Supplementary file 1 [file molecules-27-03812-s001.zip › molecules-1758241-supplementary.pdf]

Algae	Mass of raw material [g]	Mass of extract [g]	Concentration of extract in MeOH [mg/ml]	Volume of extract (added) [µl]	Mean Abs	Mean percent inhibition	mgTE/g extract	mgTE/g raw material
Cladophora aegagropila	14.996	1.026	10	50	0.645	11.568	5.344	0.366
Cladophora rivularis	2.646	0.198	10	50	0.385	47.188	16.918	1.265
Cladophora glomerata	14.922	0.355	10	50	0.396	45.725	16.442	0.391
Ulva spiralis	10.542	1.407	10	50	0.705	3.246	2.640	0.352
Ulva lactuca; Sopot	0.616	0.042	10	50	0.658	9.785	4.765	0.322
Ulva lactuca; Bornholm	1.026	0.122	10	50	0.667	8.505	4.349	0.516

Figure S1 Results of determination of the content of compounds with antioxidant activity in extracts from algae using ABTS method

Algae	Mass of raw material [g]	Mass of extract [g]	Concentration of extract in MeOH [mg/ml]	Volume of extract (added) [µl]	Mean Abs	mgFe/g extract	mgFe/g raw material
Cladophora aegagropila	14.996	1.026	10	100	0.334	1.503	0.103
Cladophora rivularis	2.646	0.198	10	10	0.083	3.580	0.268
Cladophora glomerata	14.922	0.355	10	100	0.391	1.762	0.042
Ulva spiralis	10.542	1.407	10	100	0.091	0.393	0.052
Ulva lactuca; Sopot	0.616	0.042	10	100	0.307	1.376	0.093
Ulva lactuca; Bornholm	1.026	0.122	10	100	0.171	0.758	0.090

Figure S2 Results of determination of the content of compounds with antioxidant activity in extracts from algae using the FRAP method

Algae	Mass of raw material [g]	Mass of extract [g]	Concentration of extract in MeOH [mg/ml]	Volume of extract (added) [µl]	Mean Abs	mgGAE/g extract	mgGAE/g raw material
Cladophora aegagropila	14.996	1.026	10	100	0.352	8.953	0.613
Cladophora rivularis	2.646	0.198	10	50	0.387	19.688	1.472
Cladophora glomerata	14.922	0.355	10	100	0.429	10.924	0.260
Ulva spiralis	10.542	1.407	10	100	0.06	1.437	0.192
Ulva lactuca; Sopot	0.616	0.042	10	100	0.235	5.936	0.401
Ulva lactuca; Bornholm	1.026	0.122	10	100	0.129	3.211	0.381

Figure S3 Results of determination of the content of polyphenols in extracts from algae using the Folin-Ciocalteau method

Algae	Mass of raw material [g]	Mass of extract [g]	Concentration of extract in MeOH [mg/ml]	Volume of extract (added) [µl]	Mean Abs	mgQ/g extract	mgQ/g raw material
Cladophora aegagropila	14.996	1.026	10	50	0.108	8.522	0.583
Cladophora rivularis	2.646	0.198	10	50	0.580	34.518	2.581
Cladophora glomerata	14.922	0.355	10	50	0.098	7.990	0.190
Ulva spiralis	10.542	1.407	10	50	0.025	3.957	0.528
Ulva lactuca; Sopot	0.616	0.042	10	50	0.036	4.598	0.310
Ulva lactuca; Bornholm	1.026	0.122	10	50	0.019	3.645	0.432

Figure S4 Results of determination of the content of flavonoids in extracts from algae using the complexometric with Al^{3+} *ions method*
